# Impact of small MU/segment and dose rate on delivery accuracy of volumetric‐modulated arc therapy (VMAT)

**DOI:** 10.1120/jacmp.v17i3.6046

**Published:** 2016-05-08

**Authors:** Long Huang, Tingliang Zhuang, Anthony Mastroianni, Toufik Djemil, Taoran Cui, Ping Xia

**Affiliations:** ^1^ Department of Radiation Ooncology University of Utah Salt Lake City UT USA; ^2^ Department of Radiation Oncology Clevleand Clinic Cleveland OH USA

**Keywords:** VMAT, small segment, quality assurance, delivery quality, high dose rate

## Abstract

Volumetric‐modulated arc therapy (VMAT) plans may require more control points (or segments) than some of fixed‐beam IMRT plans that are created with a limited number of segments. Increasing number of control points in a VMAT plan for a given prescription dose could create a large portion of the total number of segments with small number monitor units (MUs) per segment. The purpose of this study is to investigate the impact of the small number MU/segment on the delivery accuracy of VMAT delivered with various dose rates. Ten patient datasets were planned for hippocampus sparing for whole brain irradiation. For each dataset, two VMAT plans were created with maximum dose rates of 600 MU/min (the maximum field size of $21\times 40\;{\text{cm}}^{2}$) and 1000 MU/min (the maximum field size of $15\times 15\;{\text{cm}}^{2}$) for a daily dose of 3 Gy. Without reoptimization, the daily dose of these plans was purposely reduced to 1.5 Gy and 1.0 Gy while keeping the same total dose. Using the two dose rates and three different daily doses, six VMAT plans for each dataset were delivered to a physical phantom to investigate how the changes of dose rate and daily doses impact on delivery accuracy. Using the gamma index, we directly compared the delivered planar dose profiles with the reduced daily doses (1.5 Gy and 1.0 Gy) to the delivered planar dose at 3 Gy daily dose, delivered at dose rate of 600 MU/min and 1000 MU/min, respectively. The average numbers of segments with MU/segment≤1 were 35±8, 87±6 for VMAT‐600 1.5 Gy, VMAT‐600 1 Gy plans, and 30±7 and 42±6 for VMAT‐1000 1.5 Gy and VMAT‐1000 1 Gy plans, respectively. When delivered at 600 MU/min dose rate, the average gamma index passing rates (1%/1 mm criteria) of comparing delivered 1.5 Gy VMAT planar dose profiles to 3.0 Gy VMAT delivered planar dose profiles was 98.28%±1.66%, and the average gamma index passing rate of comparing delivered 1.0 Gy VMAT planar dose to 3.0 Gy VMAT delivered planar dose was 83.75%±4.86%. If using 2%/2 mm and 3%/3 mm criteria, the gamma index passing rates were greater than 97% for both 1.5 Gy VMAT and 1.0 Gy VMAT delivered planar doses. At 1000 MU/min dose rate, the average gamma index passing rates were 96.59%±2.70% for 1.5 Gy VMAT planar dose profiles and 79.37%±9.96% for 1.0 Gy VMAT planar dose profiles when compared to the 3.0 Gy VMAT planar delivered dose profile. When using 2%/2 mm and 3%/3 mm criteria, the gamma index passing rates were greater than 93% for both 1.5 Gy VMAT and 1.0 Gy VMAT planar delivered dose. Under a stricter gamma index criterion (1%/1 mm), significant differences in delivered planar dose profiles at different daily doses were detected, indicating that the known communication delay between the MU console and MLC console may affect VMAT delivery accuracy.

PACS number(s): 87.56.bd, 87.55.‐x

## I. INTRODUCTION

Volumetric‐modulated arc therapy (VMAT), a form of intensity‐modulated arc therapy (IMAT) introduced by Marchand et al.,^(1)^ has been increasingly used clinically to deliver intensity‐modulated radiotherapy (IMRT) plans. In VMAT, an optimized treatment plan can be delivered with either a constant or a variable dose rate and with dynamic motion of multileaf collimator (MLC) leaves while rotating the gantry at variable speeds. With the conventional fixed gantry IMRT, IMRT plans can be delivered with either the sliding window technique (DMLC)^2^, ^3^ or the step‐and‐shoot technique.^2^, ^3^, ^4^ Typically, DMLC delivery utilizes more segments with smaller monitor units (MUs) per segment than does step‐and‐shoot IMRT delivery.

During the delivery of VMAT and IMRT plans, the position of each MLC leaf is frequently monitored at a sampling rate of 50 ms for earlier models of Varian Clinax IX and Trilogy linacs (Varian Medical Systems, Palo Alto, CA) and at a sample rate of 10 ms for later models of TrueBeam linacs. The dosimetric impact of this finite sampling rate and communication delay between the MU console and MLC console resulted in segment skipping, particularly for segments with very small MUs, as discussed by Xia et al.^(4)^ and Ezzel et al.^(5)^ for DMLC and step‐and‐shoot IMRT delivery in the early Varian linac models. The dosimetric effect of such a communication delay between the MU console and MLC console on an IMRT plan depended on the delivery dose rate and the percentage of MU segments included in the IMRT plan. For the later Varian TrueBeam linac models, the communication delay has been decreased from 50 ms to 10 ms, mitigating the dosimetric effect of skipping segments.^6^, ^7^ Using the Pinnacle treatment planning system (Philips Healthcare, Andover, MA), the default number of segments for a full arc in an VMAT is typically 90 if calculated in every 4° and increases to 180 if calculated in every 2°. For a typical prostate treatment, two arcs are often used in VMAT plans to achieve a comparable or better plan quality than that of step‐and‐shoot IMRT plans. In step‐and‐shoot IMRT, the number of segments for a prostate plan is typically about 40 to 60 segments.^(8)^ Therefore, VMAT delivery may increase the number of segments, and some of these segments may be associated with small MUs, referred to as small MU segments. The dosimetric effect of the communication delay on these small MU segments of VMAT has not been reported in the literature. In this paper, we designed an experiment to study the delivery accuracy of VMAT plans and their dependence on dose rates and the number of smaller MU/segment.

## II. MATERIALS AND METHODS

### A. Treatment planning and delivery

Ten patient datasets were planned for whole‐brain irradiation with hippocampus avoidance. VMAT plans were generated according to the Radiation Therapy Oncology Group (RTOG) 0933 protocol, prescribing 30 Gy in 10 fractions. All VMAT plans consisted of a full 360° arc and a partial arc of 200° using 6 MV photons. The total number of segments for each plan was 140 (90 for 360° arc and 50 for 200° arc). The collimator angle for each plan was individually chosen from 30° to 60° based on the patient anatomy. For each dataset, two VMAT plans were optimized in the Pinnacle 9.0m at two different maximum dose rates: 600 MU/min and 1000 MU/min. For any VMAT plan created with the Pinnacle planning system, one cannot change the maximum dose rate after the plan is optimized. In order to investigate the dose rate effect on the small MU segments, we created VMAT plans for 600 MU/min and 1000 MU/min, respectively, through the inverse planning optimization with the same planning objectives. Furthermore, because the flattening filter used in 1000 MU/min beam is different from the conventional flattening filter for 600 MU/min beam, the maximum field size for 1000 MU/min beam was limited to $15\times 15\;{\text{cm}}^{2}$ and that for 600 MU/min was limited to $40\times 21\;{\text{cm}}^{2}$ for the high‐definition 120‐multileaf collimator (MLC) (the smallest leaf width of 2.5 mm). Thus, the VMAT plans using 600 MU/min dose rate cannot be directly compared to those using 1000 MU/min dose rate. Subsequently, the daily dose of these VMAT plans was purposely decreased from 3.0 Gy to 1.5 Gy and 1 Gy without reoptimization. Compared to the 3.0 Gy VMAT plans, the 1.5 Gy and the 1.0 Gy VMAT plans included more small MU segments. In the remaining manuscript, we shall refer to the VMAT plans as VMAT‐dose rate‐daily dose. For example, “VMAT‐600 1.5 Gy” represents a plan with 600 MU/min maximum dose rate and a daily dose of 1.5 Gy. A total of 60 plans (10 patient datasets, 2 dose rates, and 3 daily doses) were generated and delivered for this study. The treatment plans were delivered on a Varian Triology linear accelerator (Novalis TX). The leaf‐end position accuracy was 1.0 mm and the leaf position repeatability at isocenter was 0.6 mm.^9^, ^10^

### B. Measurements and data analysis

A MatriXX (IBA Dosimetry, Schwarzenbruck, Germany) device with 2D‐array detectors was used in this study. The $24\times 24\;{\text{cm}}^{2}$ 2D detector array consists of 1,020 parallel ion chambers with 7.65 mm separation between two detectors. Polystyrene slabs, in 11 cm and 8 cm thickness and with a physical density of 1.04 g/cm^3^, were placed on top and bottom of the ion chamber array. For each dataset, all six plans were delivered sequentially to the MatriXX device at the same position to avoid random setup uncertainty during measurements.

The number of MU/segment for each plan was exported from the Pinnacle treatment planning system (TPS). The histogram of the distribution of MU/segment for each plan was generated. The OmniPro software (IBA) was used to analyze the measured planar dose. Due to the 7.65 mm detector resolution on the MatriXX, the measured planar dose was linear‐interpolated to a resolution of 0.5 mm. For this study, the gamma index (GI) was calculated comparing the delivery dose of 3 Gy VMAT plans with the corresponding delivered dose of 1.5 Gy and 1.0 Gy VMAT plans for the given dose rate, respectively. Because of the difference dose scales among the 3 Gy, 1.5 Gy, and 1.0 Gy plans, we rescaled the delivered planar dose of 1.5 Gy plans with a factor of 2, and that of 1.0 Gy plan with a factor of 3. With this method, we removed dosimetric uncertainties associated with the treatment planning system. Three criteria of 3%/3 mm, 2%/2 mm, and 1%/1 mm were used to evaluate the delivery accuracy. Statistical analysis was performed on the passing rates of the gamma index under each criteria respectively, using the two‐way analysis of variance (ANOVA) with a significance level set as 0.05 to evaluate the impacts of either daily dose or dose rate on the gamma index.

## III. RESULTS

**Figure 1:**
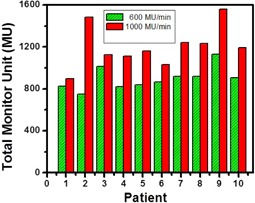
Comparison of total monitor units (MU) for 600 MU/min dose rate (green color) and 1000.

**Figure 2:**
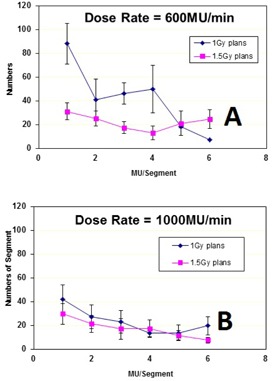
Small MU/segment distribution for VMAT‐600 plans (a) (1 Gy plan and 1.5 Gy plan) and for (b) VMAT‐1000 plans (1 Gy plan and 1.5 Gy).

**Figure 3:**
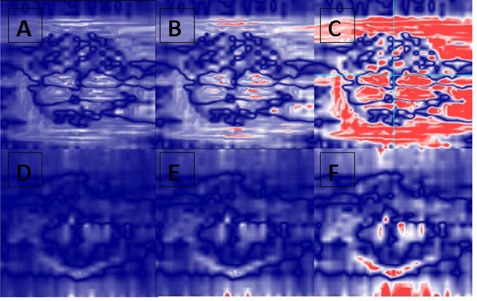
Gamma analysis of a selected VMAT plan delivered at a dose rate of 600 MU/min with 1 Gy and 1.5 Gy prescription doses compared to the prescription dose of 3 Gy at different criteria: (a) 3 mm/3% (1 Gy vs. 3 Gy); (b) 2 mm/2% (1 Gy vs. 3 Gy); (c) 1 mm/1% (1 Gy vs. 3 Gy); (d) 3 mm/3% (1.5 Gy vs. 3 Gy); (e). 2 mm/2% (1.5 Gy vs. 3 Gy); (f) 3 mm/3% (1.5 Gy vs. 3 Gy).

**Figure 4:**
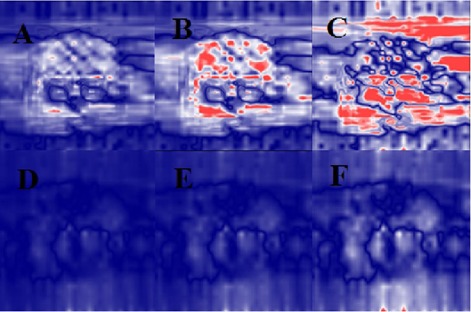
Gamma analysis of a selected VMAT plan delivered at a dose rate of 1000 MU/min with 1 Gy and 1.5 Gy prescription doses compared to the prescription dose of 3 Gy at different criteria: (a) 3 mm/3% (1 Gy vs. 3 Gy); (b) 2 mm/2% (1 Gy vs. 3 Gy); (c) 1 mm/1% (1 Gy vs. 3 Gy); (d) 3 mm/3% (1.5 Gy vs. 3 Gy); (e) 2 mm/2% (1.5 Gy vs. 3 Gy); (f) 3 mm/3% (1.5 Gy vs. 3 Gy).

The average numbers of segments with MU/segment≤1 were 35±8, 87±6, in the VMAT‐600 1.5 Gy, the VMAT‐600 1 Gy plans, and 30±7 and 42±6 in the VMAT‐1000 1.5 Gy and the VMAT‐1000 1 Gy plans, respectively. Figure 1 shows the comparison of total MU for 600 MU/min and 1000 MU/min plans. Because of different maximum field sizes for 600 MU/min and 1000 MU/min beams, the average total MUs was 898±109 in the 600 MU/min VMAT plans, lower than the average total MU of 1,203±179 in the 1000 MU/min VMAT plans. Therefore, for the same daily dose, the average number of small MU segments in the VMAT‐600 plans was higher than those in the VMAT‐1000 plans. Figures 2(a) and (b) show the distributions of the average number of MU/segment (only fewer than 7 MU/segment are displayed) in the 1.5 Gy and the 1.0 Gy VMAT plans with 600 MU/min and 1000 MU/min dose rates from the 10 datasets. As shown in Fig. 2(a), the VMAT‐600 1 Gy plans consisted of an average number of 87 segments with MUs fewer than 1, compared to an average number of 35 segments in the VMAT‐600 1.5 Gy plans. Figure 2(b) shows that the VMAT‐1000 1 Gy plans had an average number of 42 segments with MUs fewer than 1, compared to an average number of 30 segments in the VMAT‐1000 1.5 Gy plans. Figures 3(a) to 3(c) show the gamma index map for a VMAT‐600 1 Gy plan compared to the corresponding VMAT‐600 3 Gy plan using the criteria of 3%/3 mm, 2%/2 mm, and 1%/1 mm, respectively. Figures 3(d) to 3(f) show the gamma index map for a VMAT‐600 1.5 Gy plan compared to the corresponding VMAT‐600 3 Gy plan using the criteria of 3%/3 mm, 2%/2 mm, and 1%/1 mm, respectively. Similar comparisons as in Fig. 4 for VMAT plans with a maximum dose rate of 1000 MU/min are shown in Figs. 4(a) to 4(c) and Figs. 4(d) to 4(f).

Table 1 lists the gamma index passing rates for each dataset, comparing the VMAT‐600 1 Gy plans to the VMAT‐600 3 Gy plans and the VMAT‐600 1.5 Gy plans to the VMAT‐600 3 Gy plans with the criteria of 1 mm/1%, 2 mm/2%, and 3 mm/3%, respectively. In average, the passing rate of the comparisons between the VMAT‐600 1 Gy and the VMAT‐600 3 Gy plans were 83.75%±4.86%, 99.3%±0.80%, and 99.93%±0.08% under 1 mm/1%, 2 mm/2%, and 3 mm/3% criteria, respectively. A better agreement between the VMAT‐600 1.5 Gy and the VMAT‐600 3 Gy plans was observed. with the average passing rates of 98.28%±1.66%, 99.97%±0.04%, and 100%±0.00% under 1 mm/1%, 2 mm/2%, and 3 mm/3% criteria, respectively.

Table 2 lists the gamma index passing rates, comparing both the VMAT‐1000 1 Gy and the VMAT‐1000 1.5 Gy to the VMAT‐1000 3 Gy plans for each dataset under the criteria of 1 mm/1%, 2 mm/2%, and 3 mm/3%. The average passing rate were 79.37%±9.96%, 97.43%±2.67%, and 99.59%±0.52% for the VMAT‐1000 1 Gy plans, and 96.59%±2.70%, 99.92%±0.10%, and 99.99%±0.01% for the VMAT‐1000 1.5 Gy plans.

The statistical significances of gamma index passing rates of VMAT plans using different daily doses and different dose rates were tested using two‐way ANOVA. With different daily doses, there were statistically significant (p<0.05) difference on passing rate of VMAT plans. Because of different maximum field sized used for 600 MU/min and 1000 MU/min beams, the differences in gamma index passing rates for 600 MU/min and 1000MU/min VMAT plans were found statistically insignificant (p=0.15, 0.1, and 0.23 under 1%/1 mm and 2%/2 mm and 3%/3 mm criteria), albeit there was a trend that the passing rates for 1000MU/min VMAT plans were lower.

**Table 1:** Gamma indices for VMAT‐600 plans.

*Gamma Plans*	*1 Gy vs. 3 Gy*			*1.5 Gy vs. 3 Gy*		
	*1%, 1 mm*	*2%, 2 mm*	*3%, 3 mm*	*1%, 1 mm*	*2%, 2 mm*	*3%, 3 mm*
1	82.36	98.71	99.89	95.56	99.90	99.99
2	78.00	97.36	99.79	98.00	99.96	100.00
3	78.32	98.97	99.98	97.23	99.90	99.99
4	81.54	99.20	99.92	99.26	99.98	100.00
5	83.96	99.60	100.00	99.69	99.99	100.00
6	87.46	99.80	99.80	99.83	100.00	100.00
7	83.63	99.77	99.97	96.05	100.00	100.00
8	88.63	99.72	99.96	99.93	100.00	100.00
9	93.41	99.96	100.00	99.81	100.00	100.00
10	80.21	99.94	100.00	97.44	100.00	100.00
Mean	83.75	99.30	99.93	98.28	99.97	100.00
STD	4.86	0.80	0.08	1.66	0.04	0.00

**Table 2:** Gamma indices for VMAT‐1000 plans.

*Gamma Plans*	*1 Gy vs. 3 Gy*			*1.5 Gy vs. 3 Gy*		
	*1%, 1 mm*	*2%, 2 mm*	*3%, 3 mm*	*1%, 1 mm*	*2%, 2 mm*	*3%, 3 mm*
1	67.90	98.40	99.85	92.40	99.78	99.98
2	87.02	99.49	99.97	99.03	99.99	100
3	65.09	93.51	98.82	95.44	99.84	99.98
4	77.48	98.3	99.73	97.81	100	100
5	79.84	96.43	99.74	98.29	99.97	100
6	82.58	98.74	99.86	97.19	99.98	100
7	78.96	97.95	99.97	99.83	100	100
8	85.64	99.23	99.97	99.59	100	100
9	80.25	99.88	100	99.98	100	100
10	90.38	99.91	100	99.68	100	100
Mean	79.37	97.43	99.59	96.59	99.92	99.99
STD	9.96	2.67	0.52	2.70	0.10	0.01

## IV. DISCUSSION

This study demonstrated that increasing the number of small MU segments may decrease the delivery accuracy of VMAT. Other studies have also demonstrated that small MU segment (control points) may result in delivery inaccuracy in IMRT.^4^, ^11^, ^12^ For VMAT plans implemented in the Pinnacle treatment planning system, the number of segments of a VMAT plan depends on the arc length and the arc degree increment for optimization set by the users. A full arc of 360° with a 4° increment requires 90 segments (a default setting from the system, which is used in our clinic for all treatment sites although the number can be changed by the users). The number of segments would be doubled if a 2° increment were chosen for VMAT planning. If comparing the calculated dose with the delivered dose such as a typical IMRT QA, a VMAT plan calculated with 2° increment may achieve a better gamma index passing rate than that of a VMAT plan calculated with 4° increment. The trade‐off between the decreased delivery accuracy due to the increase of the number of small MU segments and increased dose calculation accuracy requires further investigation. Another confounding fact is the rescaling factor. When delivering a VMAT plan with different daily doses, this rescaling factor would change the gantry speed, MLC speed, and dose rate. This study was not designed to investigate the dosimetric effect of the changes of gantry speed and MLC speed. For a selected beam energy with a predetermined the maximum dose rate, the computer optimizer determines the instantaneous dose rate, gantry speed, and MLC speed for each segment (or two connective control points). Without reoptimization, we kept VMAT plans with different daily doses the same MLC shapes. The rescaling factor may use a lower dose rate for the reduced daily dose VMAT plans than the dose rate for VMAT plans with reoptimization. The effect of scaling factor requires further investigation.

Although the ionization chamber array was used in MatriXX to measure the absolute doses at hundreds of positions,^(13)^ Yan et al.^(14)^ showed that the strongest sensitivity to MLC position errors was to use 2%/2 mm criterion of the gamma index. Unlike the mechanical leaf position errors, which may be detected by the logfile recorded at the same rate of 50 ms or 10 ms during the communication between the MU console and MLC console, the undersampling and communication errors for small MU segments are difficult to detect in the dynalog files. Using a more stringent criterion of 1%/1 mm, we found that gamma index can detect the error due to undersampling or the communication errors of small MU segments.^(4)^ For the VMAT‐600 1 Gy plans, the mean gamma index decreased from 99.3% to 83.75% when the criterion was switched from 2%/2 mm to 1%/1 mm. For the VMAT‐1000 1 Gy plans, the mean gamma index decreased from 97.43% to 79.37% when the criterion was switched from 2%/2 mm to 1%/1 mm. Since the number of small segments increased as the daily dose decreases, we believe that the presence of small MU segments has a strong impact on gamma index passing rates, as shown in Table 1.

We also demonstrated that the dose rate may impact the VMAT plan delivery accuracy. Comparing the VMAT plans with a daily dose of 1 Gy that were created with a maximum dose rate of 600 MU/min and 1000 MU/min, the average passing rate of gamma index decreased by 4% when using the 1%/1 mm criterion, albeit not statistically significant. If the maximum field sizes were the same for both the 600 MU/min and the 1000 MU/min beams, we speculate that the decrease of gamma index in the 1000 MU/min VMAT plans would be even greater.

This study suggests that using fewer segments and lower dose rate can improve VMAT delivery accuracy. The association between the delivery accuracy and number of small MU segments and high dose rates stems from the communication delay between the MLC console and MU console. For the older models of Varian linear accelerators (prior to TrueBeam models), this communication delay is about 50 ms. With more segments with small MU, this delay may result in some of these segments being skipped.^(7)^ The effect will be amplified when a high dose rate is applied because 50 ms can deliver more MUs when a high dose rate is used. The digital model of the Varian linear accelerators (e.g., TrueBeam) decreases the communication delay from 50 ms to 10 ms, thereby reducing the likelihood of skipping segments. However, the possibility that segments may be skipped still exists in digital models of linear accelerators.

## V. CONCLUSIONS

The number of small segments included in VMAT plans may affect delivery accuracy, especially for the plans delivered with a high dose rate. Based on gamma index analysis, the criteria of 1%/1 mm gamma index can capture the difference of delivery accuracy. When using a VMAT plan prescribed to a low daily dose, the use of a very high dose rate should be done with caution.

## COPYRIGHT

This work is licensed under a Creative Commons Attribution 4.0 International License.

## Supporting information

Supplementary Material
